# The Use of Bromide of Potassium Hypodermically

**Published:** 1877-09

**Authors:** Wm. A. Adams

**Affiliations:** Bryan, Texas


### THE USE OF BROMIDE OF POTASSIUM HYPODERMICALLY.

By WM. A. ADAMS, M.D., Bryan, Texas.

It is with some reluctance that I speak of the subcutaneous use of medicine, after having read the alarming results of this mode of administration in the last issue of the Atlanta Medical and Surgical Journal.

As the danger of all theraputic agents is commensurate with the quantity, their specific therapeutic action and idiosyncrasies of different individuals; and as bromide of potass. is unprecedented for its innocence, I shall not hesitate to give my experience.

On the 18th of June, I visited a child three years of age, that was having *spasms*. On examination, and from the history of the case, I diagnosed remittent fever. They were characteristic of infantile spasms of this fever. They occurred at intervals of fifteen or twenty minutes—during the intermission vomiting was incessant. I endeavored to give bromide of potassium by the mouth as an anti-spasmodic, but in vain; I decided to use grs. v. hypodermically it being soluble in a very small quantity of water, This quantity was readily administered. My little patient had no more *spasms*. Of course, I used the magnum Dei donum, (sulph. of quinine,) as a prophylactic against the recurrence of the fever, which caused the paroxysms.

There was no unusual inflammation at the region punctured, as some times follow, this mode of giving quinine.

On the 2d of July, I had a similar case, aged seven years. I used the bromide hypo. grs. vii. with the same good effect. I have also used this method in cases of epilepsy, with not so marked effect.

Great prejudice has arisen, I fear, against this mode of administration, especially the use of morphine, from the fact that physicians are too careless about the quantity they give. Indeed I hold it is malpractice to give morphine hypodermically, without knowing accurately its weight. Patients who are not accustomed to morphine, the maximum dose to begin with should be $\frac{1}{6}$ of grain, repeated if necessary; and if this rule be observed, however marked the idiosyncrasy of your patient may be, I don't think there would arise any necessity, however, noble hearted one might be, for changing the nomenclature of diseases, for example, opium poison to "appoplexy."

